# Toward targeted therapeutics for lobular breast cancer

**DOI:** 10.1172/JCI207629

**Published:** 2026-10-01

**Authors:** Kristina A. Fanucci, Shaymaa Bahnassy, Arya Mariam Roy, Anna Sokolova, Daniel G. Stover, Peter T. Simpson, Rebecca B. Riggins, Rinath Jeselsohn

**Affiliations:** 1 Department of Medical Oncology, Dana-Farber Cancer Institute, Boston, Massachusetts, USA.; 2 Harvard Medical School, Boston, Massachusetts, USA.; 3 Department of Oncology, Lombardi Comprehensive Cancer Center, Georgetown University, Washington, DC, USA.; 4 Division of Medical Oncology, Department of Internal Medicine, The Ohio State University, Columbus, Ohio, USA.; 5 UQ Centre for Clinical Research, Faculty of Health, Medicine and Behavioural Sciences, The University of Queensland, Brisbane, Queensland, Australia.; 6 Sullivan and Nicolaides Pathology, Brisbane, Queensland, Australia.

## Abstract

Despite growing recognition of invasive lobular carcinoma (ILC) as a biologically and clinically distinct subtype of breast cancer, ILC remains understudied. Most contemporary therapeutic trials continue to enroll patients predominantly with invasive ductal carcinoma/invasive carcinoma of no special type and rarely stratify by histology. As a result, ILC’s unique disease biology, characteristic loss of E-cadherin function, diffuse growth pattern, and distinct metastatic tropism remain underrepresented in evidence that guides systemic therapy recommendations. In this Review, we examine key molecular alterations and emerging therapeutic targets in ILC, emphasizing recent preclinical discoveries that identify subtype-specific therapeutic vulnerabilities and guide the development of histology-specific treatment approaches for this unique disease. In combination with endocrine therapies, effective targeting in ILC may require a multilayered strategy that extends beyond genomic alterations to leverage ILC’s specific estrogen receptor–associated proteins, metabolism, and tumor microenvironment. Future clinical trial frameworks incorporating prespecified ILC cohorts, tailored endpoints, and coclinical approaches enabling parallel testing in patients and patient-derived models could help accelerate the development and evaluation of ILC-targeted therapeutics.

## Introduction

Invasive lobular carcinoma (ILC) represents 10%–15% of invasive breast cancers and exhibits unique biological, molecular, and clinical features that distinguish it from invasive ductal carcinoma/no special type (IDC/NST) (1, 2). Characterized by its loss of function of the cell adhesion molecule E-cadherin (2), ILC often presents with a more diffuse infiltrative pattern rather than a discrete mass (3), which can lead to challenges in detection (4, 5). It also has unique patterns of presentation and metastatic spread that distinguish it from NST (6–9). The majority (90%–95%) of ILC is estrogen receptor positive (ER^+^) (10, 11), and survival is slightly higher than for NST during the first five years following diagnosis, but rates of recurrence and death after five years are higher in patients with ILC (10, 12). Despite these important differences, patients with ILC are often underrepresented in clinical trials, and explicit analyses of treatment benefit in patients with ILC are rare. Consequently, no treatment strategy has been specifically established for this distinct histological breast cancer subtype.

In this Review, we highlight key molecular alterations and emerging therapeutic targets in ILC, emphasizing recent preclinical advances to uncover subtype-specific therapeutic vulnerabilities and promising paths toward treatments designed for this unique breast cancer.

## Leveraging ILC genomic alterations for targeted therapies

Therapies directed at common ILC genomic alterations offer opportunities to refine systemic treatment strategies. Studies have described the most common alterations in both the early-stage and metastatic ILC settings (13–17). Compared with NST, ILC shows higher frequencies of Cadherin 1 (*CDH1*) alterations, phosphatidylinositol 3-kinase catalytic subunit alpha (*PIK3CA*) mutation, and Erb-B2 receptor tyrosine kinase 2 (*ERBB2*) mutations, regardless of stage (13–16). *FOXA1* mutations are more common in ILC than NST in primary and metastatic disease while *TP53* mutations are less common than in NST (13–17). The metastatic setting of ILC is further characterized by *ESR1* mutations occurring at a meaningful frequency (16).

### CDH1 alterations.

While alterations other than E-cadherin loss can potentially result in ILC phenotypes of cellular dissociation (18–20), loss of E-cadherin due to truncating mutations or loss-of-function alterations in *CDH1* is the hallmark of ILC. Although not directly druggable, the loss of E-cadherin creates the disease-defining biology and provides potential therapeutic strategies.

Upon loss of E-cadherin, cytosolic p120-catenin (p120) shifts from a membranous to a cytosolic localization, and β-catenin is mostly lost (21). In a mouse model of ILC it was shown that cytosolic p120 interacts with and inhibits the Rho/Rock antagonist myosin phosphatase Rho-interacting protein, resulting in increased Rho/Rock signaling and leading to anchorage-independent growth of the tumor (21). Increased nuclear p120 has also been observed in models of ILC, resulting in decreased repression of Kaiso-mediated transcription leading to upregulation of Wnt11, subsequent RhoA activation, and resistance of ILC cells to anoikis (22, 23). Rho kinase inhibitors are used clinically in the treatment of graft-versus-host disease and for prevention of cerebral vasospasm, and a preclinical study has shown fasudil, a Rho kinase inhibitor, decreased tumor growth in three mouse models, including a breast cancer model (24). Inhibitors of these pathways are putative targets for investigation in patients with ILC.

Multiple synthetic lethality screens have exploited this vulnerability and identified drug candidates with pronounced inhibitory effects in *CDH1-*deficient cells (25). These include p21-activated kinase, phosphoinositide 3-kinase (PI3K)/mechanistic target of rapamycin (mTOR), c-ros avian UR2 sarcoma virus oncogene homolog 1 (ROS1)/anaplastic lymphoma kinase (ALK)/Mesenchymal Epithelial Transition proto-oncogene receptor tyrosine kinase (MET) inhibitors (26). One of the strongest hits in the synthetic lethality screens was the ROS1 RTK (26), a proto-oncogene involved in cell survival, growth, and proliferation (27). Experimental validation in E-cadherin–deficient preclinical models confirmed E-cadherin/ROS1 synthetic lethality (26). Promising preclinical testing of ROS1 inhibition induced selective cell death in *CDH1*-mutated cell lines by promoting cytokinesis failure, potentially by downregulating p120 (26). This ROS1 inhibition testing, however, was limited to a single ER^+^ ILC cell line and to animal models that do not capture ILC’s ER dependence and are therefore not typical of ILC, raising questions about translatability of these findings.

Two clinical trials, ROLo and ROSALINE, tested ROS1-targeted agents (e.g., crizotinib, entrectinib; Table 1) in ILC. In the ROLo trial, patients with recurrent, inoperable, locally advanced ER^+^/HER2-negative (HER2^–^) ILC received crizotinib and the selective estrogen receptor degrader (SERD) fulvestrant, with an objective response rate (ORR) of 4% and a median progression-free survival (PFS) of 1.8 months (28). In the ROSALINE trial, patients with stage IIA–IIIA hormone receptor–positive (HR^+^)/HER2^–^ ILC were treated in the neoadjuvant setting with the aromatase inhibitor letrozole and entrectinib. No evaluable patients achieved a residual cancer burden of 0 or 1, and 1 (2%) nonevaluable patient achieved a pathologic complete response (29). While neither the ROLo nor ROSALINE trials met their primary endpoint, the neoadjuvant ROSALINE trial showed some evidence of radiological activity of endocrine therapy (ET) and entrectinib, with an ORR of 49% by MRI (28, 29). Meanwhile, the REPLOT phase II trial (NCT06408168), which was investigating the next-generation ROS1 inhibitor repotrectinib given alone or with fulvestrant in ER^+^/HER2^–^ metastatic ILC, was terminated early.

The discordance between promising preclinical findings and limited clinical efficacy of ROS1 inhibition raises the question of whether the drugs tested were sufficiently potent ROS1 inhibitors at a tolerable dose and call the validity of the target itself into question. The results also highlight the need to validate preclinical findings in ILC-specific models and to confirm expression of potential ILC targets in patient tumor samples. However, assessing ROS1 expression by immunohistochemistry in breast tumor samples is complicated by high antibody clone dependence (30), and whether expression differs in ILC versus NST or primary versus metastatic ILC remains unclear.

Loss of function of E-cadherin also increases dependence on the alternate pathway, proto-oncogene tyrosine-protein kinase SRC, a non-RTK, for proliferation (31). Targeting this vulnerability, bosutinib — a tyrosine kinase inhibitor of SRC and BCR-ABL approved for treatment of chronic myelogenous leukemia — has been evaluated in HR^+^/HER2^–^ metastatic breast cancer. A phase I trial (NCT03854903) showed that the combination of bosutinib, fulvestrant, and the cyclin-dependent kinase 4 and 6 inhibitor (CDK4/6i) palbociclib was well tolerated and had an encouraging clinical benefit rate of 50% at 6 months (32) (Supplemental Table 1; supplemental material available online with this article; https://doi.org/10.1172/JCI207629DS1).

### Alterations in the PI3K/AKT/mTOR pathway.

PI3K/AKT/mTOR pathway alterations are frequent in ILC and found in 35%–60% of patients with this histology (13–16). In addition, loss of function of E-cadherin can contribute to loss of phosphatase and tensin homolog (*PTEN*) expression and result in aberrant activation of PI3K/AKT signaling through increased responsiveness to autocrine growth factor receptor–dependent activation (33–37). This inherent vulnerability suggests a potential increased benefit of inhibition of this pathway in patients with ILC independent of mutation status. Progesterone receptor negativity, which has been associated with decreased endocrine sensitivity and is reported in about 21% of patients with metastatic ILC, has been found to be associated with increased activity of the PI3K/AKT/mTOR pathway (16, 38). Agents targeting this pathway (e.g., PIK3CA inhibitors: alpelisib, inavolisib; AKT inhibitor: capivasertib; mTOR inhibitor: everolimus; pan-PI3K/mTOR inhibitor: gedatolisib) are approved for metastatic HR^+^/HER2^–^ breast cancer and are used following progression on first-line CDK4/6i or in the first-line setting for patients who progress on or shortly after completing adjuvant ET (Supplemental Table 1). The LOBSTER trial, a neoadjuvant trial of fulvestrant versus capivasertib plus fulvestrant, is enrolling exclusively patients with primary high-risk ILC regardless of mutations in the PI3K/AKT/mTOR pathway (NCT06607757) (Table 1).

Although trials have not been powered for histology-specific comparisons of outcomes, real-world retrospective analyses have examined outcomes in patients with ILC versus NST treated with agents targeting this pathway. A retrospective study by Mouabbi et al. reported that the addition of targeted therapies such as the PIK3CA inhibitor alpelisib or mTOR inhibitor everolimus to ET resulted in comparable PFS and overall survival (OS) outcomes in patients with HR^+^/HER2^–^ metastatic ILC versus NST (39). Ongoing trials of PI3K/AKT/mTOR pathway inhibitors in different combination regimens for *PIK3CA*-mutant breast cancer — in both early-stage (NCT05306041) and advanced settings (40) — and for patients without *PIK3CA* mutations in the advanced setting (41) may hold particular benefit for those with ILC. However, the number of patients with ILC included in these trials and whether there will be sufficient statistical power for future retrospective subgroup analyses remain unclear.

### ESR1 mutations.

Estrogen receptor 1 *(ESR1)* mutations are acquired under the selective pressure of ET and result in constitutively active estrogen signaling (42). These mutations have been described in 10% of patients at the time of initial diagnosis with metastatic ILC (16) and in up to 60% of patients after progression on ET (42). Trials of oral SERDs (e.g., elacestrant, giredestrant, imlunestrant) have shown the greatest benefit in patients with *ESR1*-mutant disease (Supplemental Table 1). Next-generation ETs (e.g., SERDs, complete estrogen receptor antagonists, proteolysis-targeting chimeras), especially in combination with other targeted and standard-of-care therapies, are being evaluated in trials for patients with ER^+^ disease, including patients with ILC, and may offer meaningful therapeutic benefit.

### ERBB2 alterations.

In ILC, *ERBB2* amplification resulting in classical HER2^+^ disease is rare, but *ERBB2* mutations are enriched and associated with high recurrence scores and resistance to ET (43). Alterations in *ERBB2* are present in 4%–5% of early-stage ILC tumors (13–15) and 6%–27% of tumors from patients with metastatic ILC (16, 44, 45). In a cohort of *CDH1*-mutated recurrent ILC, Ross et al. identified 6/22 (27%) cases with *ERBB2* alterations: 4 (18%) with mutation, 1 (5%) with gene fusion, and 1 (5%) with amplification (44). HER2 is therefore a clinically important target in ILC, with trials showing benefit of drugs targeting this protein (e.g., trastuzumab, trastuzumab-deruxtecan, neratinib) (Supplemental Table 1). The SUMMIT trial enrolled patients with HR^+^/HER2^–^ metastatic breast cancer with activating *ERBB2* mutations. Similar to NST, patients with ILC treated with the tyrosine kinase inhibitor neratinib, fulvestrant, and HER2 monoclonal antibody trastuzumab were found to have a clinically meaningful median PFS of 8.3 months (46). Likewise, the DESTINY-PanTumor01 trial enrolled patients with metastatic solid tumors with activating *ERBB2* mutations and established the benefit of trastuzumab-deruxtecan in this population (47). The DESTINY-Breast06 trial enrolled patients with HR^+^/HER2-low or -ultralow disease and randomized them to receive either the antibody-drug conjugate (ADC) trastuzumab-deruxtecan or physician’s choice chemotherapy. They showed improvement in median PFS of 13.2 months in the trastuzumab-deruxtecan group versus 8.1 months in the chemotherapy group, establishing HER2-low and -ultralow as important biomarkers in the treatment of HER2^–^ breast cancer (48). HER2-low expression has been reported in 40.6%–57% of patients with early-stage ILC (49–51) and 91.6% of patients with metastatic ILC at the time of autopsy though with varying consistency across metastatic sites (7.5%–100%) (52). In the neoadjuvant setting, neratinib is being studied in an actively recruiting ILC-specific trial (NCT05919108) for patients with stage I–III *ERBB2*-mutated ILC. Participants will be treated with neratinib for 24 weeks before surgery, and the preoperative endocrine prognostic index score will serve as the primary endpoint for this trial. Beyond *ERBB2* mutations and given the demonstrated benefit of HER2-directed therapies in patients with low and ultralow HER2 expression (48), investigation of potential benefit of other HER2-targeting agents in ILC is warranted.

### ERBB3 alterations.

*ERBB3* alterations have been reported in 4% of patients with ILC and in 14% of patients with early-stage disease and a high LobSig score, a 194-gene signature developed for prognostication in ILC (13, 53). This finding raises the possibilities of increased sensitivity of ILC to HER3-directed therapy such as patritumab deruxtecan and of the potential benefit of agents targeting this protein in the early- and late-stage settings (54) (Supplemental Table 1).

### FGFR alterations.

*FGFR* alterations, including amplifications and mutations, are linked to aggressive disease, endocrine resistance, and recurrence in ILC, contributing to poorer clinical outcomes. In particular, *FGFR4* is overexpressed in endocrine-resistant cell lines, and both high expression and hotspot mutations (3.5% in ILC vs. 0.5% in NST) are observed in endocrine-treated metastatic ER^+^ breast cancer (55). The *FGFR4* activity gene signature is also markedly upregulated post-neoadjuvant aromatase inhibitor treatment, and high FGFR4 expression predicts worse distant recurrence-free survival in patients with ER^+^ ILC (56). However, in ILC cell models, *FGFR4* knockdown did not alter response to endocrine treatment, nor did its pharmacological inhibition synergize with fulvestrant treatment (56). This raises questions about the potential clinical utility of FGFR4 inhibition for patients with ER^+^ ILC.

Conversely, family member FGFR1 drives endocrine resistance through activation of MAPK and PI3K signaling (57). Amplification of *FGFR1* has been reported in 25% of early-stage and 7% of metastatic ILC, whereas *FGFR1* point mutations are less common (13, 16). While a prior in-depth analysis of chromosome 8p11-12 amplifications, which includes the FGFR1 locus, demonstrated that FGFR1 was not the driver of this amplicon (58), inhibition of FGFR1 reduced viability of an ILC cell line (59). Several inhibitors of FGFR (e.g., ADZ4547, erdafitinib, Debio 1347) have been developed and show modest effect in breast cancer trials (Supplemental Table 1). Results are awaited from an ongoing phase Ib/II study of lenvatinib, a multikinase inhibitor with activity against FGFR1–4, in combination with pembrolizumab and fulvestrant in ER^+^/HER2^–^ metastatic breast cancer (NCT06110793). Future trials investigating pan-FGFR or FGFR1-selective agents in combination with ET or medications targeting the PI3K/AKT pathway may be beneficial.

### FOXA1 mutations.

*FOXA1* mutations have been reported in 7%–9% of early-stage and metastatic ILC (13, 14, 16). This pioneer transcription factor is of particular interest in ILC. Preclinical work has shown that gain of FOXA1 recruitment to chromatin generates an ILC-specific FOXA1/ER axis that promotes transcription of genes associated with progression (60). FOXA1 modulates ER binding and hormone responsiveness (61), and its expression, along with GATA-binding protein 3 (GATA3), closely associates with ER/*ESR1* expression status (62, 63). The higher rate of *FOXA1* mutations and lower rate of *GATA3* mutations in ILC than in NST (14, 16) highlights the unique ER axis and suggests a potentially different response to ET in ILC.

Retrospective studies have shown relative tamoxifen resistance in patients with ILC (64), which may be explained by the sustained ER chromatin binding because of the distinct chromatin state of the FOXA1/ER axis (60). Additionally, targeted silencing of a unique FOXA1 regulatory binding site inhibited ILC cell growth (60). Collectively, these findings support the use of aromatase inhibitors and SERDs over tamoxifen in ILC and identify FOXA1 as a potential therapeutic target for future investigation, with small molecule inhibitors of FOXA1/2 being investigated in preclinical studies (65, 66).

### CCND1 amplification.

*CCND1* encodes cyclin D1, which forms a complex with CDK4/6 to drive the G1-to-S transition of the cell cycle, and amplification of this gene is common in ILC (38% in early-stage ILC, 16% in metastatic ILC) (13, 16). A preclinical study in mouse and organoid models of ILC showed inhibitor of DNA binding 2 (Id2), a gene important in cell cycle progression, is critical for anoikis resistance and that E-cadherin loss increases expression of Id2. Id2 levels were found to be increased in ILC compared with other subtypes of breast cancer, and Id2 accumulation in the cytosol resulted in G0/G1 cell cycle arrest that was CDK4/6 dependent, suggesting Id2 acts as an antagonist of CDK4/6 by binding to hypophosphorylated Rb (20). This may lead to cyclin D1 upregulation and increased sensitivity to CDK4/6i in ILC. CDK4/6i (e.g., ribociclib, abemaciclib, palbociclib) remain standard first-line treatment for patients with ER^+^ metastatic breast cancer including those with ILC and are an important component of adjuvant treatment for patients with high-risk, early-stage disease. Preclinical studies found particular sensitivity of luminal ER^+^ cell lines to CDK4/6i, which defines most ILCs (67, 68). An exploratory analysis of patients with advanced HR^+^/HER2^–^ ILC in the MONALEESA-3 trial receiving first- or second-line treatment showed a median PFS of 20.5 months with fulvestrant and ribociclib compared with 9.4 months with placebo (hazard ratio = 0.56, 95% CI 0.37–0.86), providing evidence for benefit in this subgroup (69). The PENELOPE-B trial compared adjuvant standard ET with or without one year of palbociclib in patients with high-risk HR^+^/HER2^–^ breast cancer who had residual disease after neoadjuvant chemotherapy. While no statistically significant benefit was seen in the overall population, exploratory subgroup analysis suggested potential improved invasive disease–free survival and OS in patients with ILC treated with palbociclib (70). These findings provide encouraging evidence that CDK4/6i standard treatments can benefit patients with ILC. Outcomes for patients with ILC who are included in trials of triplet therapies that include a CDK4/6i are anticipated (40) (NCT04862663, NCT07085767, NCT05608252, NCT06726148, NCT06239467).

## Other emerging tumor-intrinsic therapeutic targets for ILC

With the growing recognition of ILC as a distinct entity among breast cancers, ongoing basic and translational research is uncovering potential ILC-specific targets across tumor-intrinsic pathways (Figure 1).

### ILC-specific ER-associated proteins.

Given that the vast majority of ILCs (90%–95%) are ER^+^ (10, 11), targeting ILC-specific, ER-interacting proteins or disrupting their unique assemblies could represent a promising therapeutic avenue to enhance endocrine responsiveness. Proteomic profiling of nuclear ER-associated proteins identified ILC-specific binding partners distinct from NST (71), and among those are at least two novel ER-coregulator complexes with gene-regulatory functions extending beyond their canonical roles.

For example, knockdown of mediator of DNA damage checkpoint 1 (MDC1) suppresses ILC cell proliferation and endocrine resistance while dysregulating the ER transcriptome, revealing a novel ILC-specific ER-MDC1 interaction that drives ER-dependent proliferation and survival while compromising MDC1’s canonical DNA repair function (71, 72). This interaction generates a “BRCA-like” phenotype in ILC cells in the absence of canonical breast cancer gene (*BRCA*) mutations, with dysfunctional DNA repair evidenced by delayed initiation and impaired resolution of DNA double-strand breaks following ionizing radiation. The ER-MDC1–mediated gene regulation activity is also associated with upregulation of poly (ADP-ribose) polymerase–mediated (PARP-mediated) repair pathways, and treatment with the FDA-approved PARP inhibitor talazoparib, alone or in combination with ET, produced durable growth suppression in ILC preclinical models, even after treatment cessation (72). Together, these findings highlight the ER-MDC1 axis as a compelling target that could enhance endocrine responsiveness while sensitizing ILC cells to PARP inhibition or radiation therapy (72, 73).

Glutamate dehydrogenase 1 (GLUD1), a metabolic enzyme that catalyzes the interconversion of glutamate and alpha-ketoglutarate, emerges as another promising target, with elevated expression correlating with larger primary tumors and uniquely associated with ER expression in patients with ILC but not NST. Pharmacological inhibition of GLUD1 reduces ER expression and cell viability, even in ET-resistant ILC cells, where it is upregulated (74). The correlation between GLUD1 and ER expression (74), together with evidence from noncancerous contexts of a nuclear GLUD1 subpopulation involved in chromatin remodeling and transcription (75, 76), suggests a nuclear role of GLUD1 in modulating ER activity. However, its precise ER-coregulatory function in ILC remains to be determined, and whether its inhibition can potentiate growth-suppressive effects of ET in vivo requires investigation.

### Metabolic targets.

Mounting evidence from clinical imaging and preclinical studies indicate that ILC exhibits a distinct metabolic phenotype from that of NST, characterized by reduced glucose dependence and increased reliance on lipid and amino acid metabolism (77–83). In patient tumors, a network of four closely related glutamate-handling proteins correlates with adverse clinicopathological features in ILC but not NST (74). Deregulation of amino acid metabolism is markedly pronounced in ET-resistant ILC, especially within the glutamate and aspartate pathways (84), and is accompanied by altered expression of metabolic regulators and enzymes. Among these, GLUD1 and glutathione peroxidase 4 (GPX4) are upregulated (74), while mutations in metabotropic glutamate receptors (GRMs or mGluRs) have been identified in ET-resistant models (85). Global glutamate blockade with FDA-approved riluzole suppressed proliferation and restored ET sensitivity in multiple preclinical models, including ET-resistant ones (86, 87). Targeted inhibition of individual network components — especially GLUD1 and GPX4 — requires further in vitro and in vivo evaluation to assess their therapeutic potential in ET-resistant disease.

In tamoxifen-resistant (TamR) ILC cells, low argininosuccinate synthase (ASS1) expression — caused by methylation-mediated silencing of *ASS1* — diverts aspartate toward nucleotide biosynthesis, fueling proliferation. Low ASS1 expression correlated with decreased OS in ET-treated patients with ILC, as shown in subtype-specific analyses of the METABRIC dataset (84). Restoration of ASS1 expression using decitabine, a demethylating drug, or inhibition of pyrimidine biosynthesis with farudostat improved tamoxifen sensitivity and suppressed proliferation of TamR cells (84). While these findings highlight ASS1 as a potential biomarker and therapeutic target, further validation of its reduced protein-level expression in patient tumors and in vivo testing of these interventions remain essential to translate these insights into therapies for endocrine-resistant ILC.

Beyond amino acid metabolism, ET-resistant ILC also exhibits dysregulated lipid metabolism, including upregulated aldo-keto reductase family members (AKR1C1/2/3) and SREBP1. Genetic or pharmacological inhibition of these targets in ET-resistant ILC cells suppressed their growth or restored tamoxifen sensitivity (88, 89) (reviewed in ref. 90), which highlights exploitable vulnerabilities. Together, members of glutamate, aspartate, and lipid pathways could represent actionable targets for precision therapies, especially in ET-resistant ILC.

### Signaling pathways.

Signaling pathways enriched in ILC may represent exploitable therapeutic vulnerabilities. In a recent study, in silico transcriptomic analysis of three large breast cancer datasets identified a relative increase in cAMP/PKA/CREB signaling in ILC compared with NST tumors, which was confirmed in multiple in vitro models (91). The cAMP/PKA/CREB pathway regulates gene expression and has been found to either promote or suppress tumor growth, depending on tumor type and context (92). Pharmacological activation of this pathway increased intracellular cAMP and caused greater growth inhibition in ILC versus NST cell lines (91). These findings suggest a potential role for cAMP modulators as a targeted treatment approach in ILC and warrant future in vivo investigation.

### Epigenetic regulators.

The bromodomain and extra-terminal domain (BET) family of proteins, epigenetic readers that regulate gene transcription by binding acetylated histones, are a rational therapeutic target for ILC (93). This is supported by clinical evidence of the association between high expression of BET genes *BRD3/BRD4* and poor survival specifically in patients with ILC (93). In addition, in preclinical studies the BET inhibitor JQ1 inhibited growth in all four tested ILC/ILC-like cell lines and induced apoptosis in two models. Increased expression of FGFR1 was identified in the two cell lines demonstrating resistance to the cytotoxic effects of JQ1, and the addition of an FGFR1 inhibitor resulted in sensitivity to JQ1. The finding of *FGFR1* amplifications in patients with early and metastatic ILC (discussed above) strengthens the translational rationale for testing the combination of BET and FGFR1 inhibitors, especially in recurrent (93) or advanced disease settings.

### Targets for ADCs.

ADCs are a fast-growing class of targeted therapies consisting of a recombinant monoclonal antibody bound to cytotoxic payload via a linker molecule (94). Four ADCs have approval for use in breast cancer (sacituzumab-govitecan, trastuzumab-deruxtecan, datopotamab-deruxtecan, and trastuzumab-emtansane) (48, 95–97). Investigating postmortem samples from patients with metastatic breast cancer, TROP2, B7-H4, NECTIN4, HER2, and HER3 were noted to have higher expression in tumor compared with normal tissue, making them potential targets for ADCs (95). While there were no statistically significant differences between samples from patients with NST and ILC, B7-H4 expression was seen numerically more often in metastatic samples from patients with ILC, and ADCs targeting B7-H4 may be of particular interest in patients with ILC (94).

## Emerging tumor-extrinsic therapeutic targets

ILC is increasingly recognized as a disease profoundly shaped by its tumor microenvironment (TME). While intrinsic factors such as *CDH1* loss and distinct endocrine biology contribute to its unique phenotype, the tumor-extrinsic ecosystem plays an equally critical role in determining ILC progression, dissemination, therapeutic vulnerabilities, and metastatic behavior (Figure 1). Recent integrative analyses, including single-cell RNA sequencing, spatial transcriptomics, and functional modeling, have revealed that the stromal and immune compartments of ILC differ markedly from those of NST, suggesting that ILC evolves within a highly specialized microenvironment (98, 99).

### Cancer-associated fibroblast–dependent vulnerabilities.

Across multiple independent public datasets, ILC demonstrates a distinct enrichment of specialized cancer-associated fibroblast (CAF) subsets, often with a more pronounced stromal signature than NST (100). These CAFs orchestrate several paracrine programs that influence tumor behavior (101). One of the most prominent pathways involves IL-6–mediated activation of JAK/STAT3 signaling in ILC cells, which enhances tumor cell motility, promotes partial epithelial-mesenchymal transition (EMT) phenotypes, and suppresses ER target gene expression (100). This IL-6/STAT3 axis contributes to both early dissemination and endocrine resistance, a clinically relevant phenomenon given the often-late recurrences observed in ILC (100, 102). Together, these findings suggest that stromal signaling, rather than tumor-intrinsic alterations alone, may drive therapeutic escape in ILC.

In addition, CAFs in ILC show enriched expression of extracellular matrix–regulating (ECM-regulating) enzymes and IGF-modulatory proteins such as pregnancy-associated plasma protein-A, which increases IGF-1 bioavailability, supporting downstream prosurvival signaling (103, 104). These interactions collectively position CAFs not merely as supportive stromal cells but also as active drivers of key biological features unique to ILC. Consequently, CAF-directed therapies, including IL-6/STAT3 inhibitors, fibroblast activating protein–targeted (FAP-targeted) agents, and IGF pathway modulation, are emerging as a rational therapeutic strategy that warrants further investigation in combination with ET. In particular, FAP is emerging as a promising diagnostic and therapeutic target, though evidence remains confined to studies with small patient numbers. FAP inhibitor PET/CT was superior to traditional ^18^F-FDG (fluorine-18 fluorodeoxyglucose) PET/CT for detecting infiltrative soft tissue and serosal metastases in patients with ILC (105). Several FAP-targeted theranostic radiotracers have been developed and clinically tested across multiple tumor types (106–108), and these hold promise as a future therapeutic option for patients with ILC.

### ECM remodeling.

ILC is characterized by a distinct stromal architecture marked by collagen-rich matrices, altered ECM alignment, and mechanical cues that facilitate its classical “single-file” infiltration pattern. The loss of E-cadherin function forces ILC cells to rely more heavily on integrin-mediated adhesion and mechanotransductive signaling for migration and survival (109). Studies have demonstrated that collagen density, fiber orientation, and tensile properties guide directional motility in ILC more strongly than in NST (110).

Enzymes such as lysyl oxidase (LOX) and MMPs reshape the ECM to create permissive tracks for local invasion. LOX-1 and LOX family proteins are overexpressed in breast cancer and play an important role in ILC by regulating ECM remodeling. LOX-mediated collagen and elastin cross-linking increases matrix stiffness, facilitating tumor growth, invasion, and metastasis (110–113). In parallel, LOX-1 signaling activates oncogenic pathways, including PI3K/AKT and EMT, promoting tumor cell survival and motility and correlating with adverse clinical outcomes (114).

Preclinical studies suggest that ILC cells develop a functional dependence on lysyl oxidase like 1-mediated) (LOXL1-mediated) ECM remodeling, relying on the altered mechanical and biochemical properties of the TME for survival and proliferation. By modifying collagen and elastin fibers, LOXL1 supports the diffuse, single-file invasion pattern characteristic of ILC and facilitates metastatic spread to collagen-rich distant sites. This ECM dependency creates a context in which LOXL1 inhibition disrupts tumor-stroma crosstalk, impairing tumor growth and viability (115, 116). Targeting LOXL1, either alone or in combination with ET, PI3K/AKT pathway inhibitors, or emerging stromal modulating strategies, represents a promising avenue to disrupt the supportive tumor niche and overcome therapeutic resistance in ILC (110, 116). Furthermore, changes in collagen fiber matrices (density/alignment) and MYC/AP-1 gene signatures have been proposed as sensitive readouts to evaluate response to LOX-targeted therapies in future ILC window-of-opportunity trials (117).

### Adipocyte-tumor crosstalk and metabolic dependencies.

ILC demonstrates a pronounced tropism for adipose-rich metastatic sites such as the peritoneum, omentum, and gastrointestinal tract (90). Adipocytes surrounding ILC lesions can transfer fatty acids and metabolic substrates to tumor cells, supporting oxidative metabolism and growth (118). Although most mechanistic work on adipocyte–breast cancer interactions is derived from NST, emerging evidence indicates that ILC cells may be particularly dependent on fatty acid uptake and oxidation pathways, potentially explaining their metastatic preferences (119). Therapeutic strategies targeting this metabolic crosstalk, including fatty acid oxidation inhibitors (e.g., carnitine palmitoyltransferase 1 inhibitors, fatty acid binding protein 4 blockade, and metabolic reprogramming agents), may offer new opportunities for ILC-specific interventions.

### Tumor immune microenvironment.

The immune microenvironment of ILC is characterized by a predominantly immune-cold phenotype, distinguished by low tumor-infiltrating lymphocytes (TILs) (median TILs 5%, 15% of ILC with TILs > 10%), sparse cytotoxic T cell activity, and limited interferon signaling relative to NST (98, 120, 121). While transcriptomic profiling sometimes reveals *higher* immune-related gene expression in ILC, spatial biology demonstrates that immune cells often accumulate in the stroma rather than infiltrating tumor nests, generating an immune-excluded architecture that restricts effective antigen recognition (98, 122). Stromal IL-6–driven STAT3 activation in the TME further reinforces immunosuppression by reducing antigen presentation and impairing effector T cell recruitment (123, 124). A subset of ILC tumors identified as immune related by bulk and spatial transcriptomic profiling is characterized by macrophage-associated signaling, lymphocyte infiltration, and increased expression of checkpoint proteins (14, 15, 99) and has unique clinical outcomes compared with other ILC transcriptional subtypes (14, 99). Moreover, analysis of the TME in primary ILC revealed programmed cell death ligand 1 (PD-L1) expression on both tumor cells and TILs in a subset of samples, suggesting both adaptive and constitutive PD-L1 expression in some tumors and motivating future exploration of the potential benefit of immunotherapy in select patients with ILC (125).

Despite this overall immune-cold phenotype, a clinically meaningful minority of ILCs exhibit high tumor mutational burden (TMB) or underlying genomic instability, occasionally associated with apolipoprotein B mRNA editing enzyme, catalytic polypeptide (*APOBEC*) mutagenesis or homologous recombination deficiency (126). High-TMB (≥10 mutations/Mb; ~18%) ILCs show increased neoantigen load, greater immune infiltration, and in select cases, clinically observed durable responses to immune checkpoint inhibitors (127–129). Although these represent a small subset, their existence underscores the biological diversity within ILC and highlights the value of molecular profiling to identify patients who may benefit from immunotherapy.

Given the predominant immune-excluded phenotype, successful immunotherapy in ILC will likely require combination approaches that modify stromal or metabolic barriers, such as IL-6/STAT3 pathway inhibition, CAF modulation, ECM clearance, or targeting immunometabolic interactions in adipocyte-rich tissues. In select cases, combining immune checkpoint blockade with immunogenic chemotherapy, as shown in the GELATO trial (NCT03147040) (Table 1), may represent a promising strategy to overcome immune exclusion, especially for patients with triple-negative metastatic ILC (130). Together, these insights indicate that while ILC is broadly immune cold, it is not immunologically uniform, and biomarker-driven patient selection will be critical for effective immunotherapy use.

## Considerations to enable ILC-specific targeted therapeutic strategies

Several key opportunities exist to improve clinical trial design and ensure that therapeutic strategies are appropriately evaluated for the ILC population. Future clinical trials in ILC must move beyond histology-agnostic designs to better capture subtype-specific therapeutic signals. Histology-based eligibility criteria, minimum ILC representation, or dedicated ILC cohorts are needed, as pooling ILC with NST may obscure meaningful differences driven by distinct genomic alterations, lower proliferative indices, and unique therapeutic vulnerabilities in ILC (131). Endpoints should also be tailored to ILC’s clinical behavior, which is characterized by indolent growth, late recurrences, and nonvisceral metastatic patterns. Traditional short-term radiographic response metrics may underestimate benefit, highlighting the importance of incorporating long-term PFS, clinical benefit rates, and patient-reported outcomes that reflect symptom burden from gastrointestinal and peritoneal disease (13, 131).

Trial design should additionally account for ILC-specific imaging, biomarker, and immune characteristics. Given ILC’s diffuse infiltration and low FDG avidity, advanced imaging modalities and circulating tumor DNA monitoring may provide more sensitive assessments of disease burden and treatment response than conventional response evaluation criteria in solid tumors (RECIST) criteria (132). In parallel, other emerging multimetric readouts, as shown by Flaherty et al. (117), may serve as new surrogate endpoints and offer additional ways to assess response to targeted therapies in future trials. Although most ILC tumors exhibit an immune-cold microenvironment with low TIL density, a subset demonstrates high TMB and clinically meaningful responses to immune checkpoint blockade, underscoring the need for biomarker-driven enrichment strategies rather than histology alone. Finally, adaptive, umbrella, and platform trial designs incorporating biomarker-defined ILC subgroups offer an efficient path to evaluating emerging therapies — including oral SERDs, PI3K/AKT inhibitors, and novel endocrine combinations — while integrating patient-centered and metastatic site–specific outcomes relevant to this distinct disease subtype.

Designing experimental studies that increasingly mirror clinical trial structure, as shown in Flaherty et al. (117), and implementing innovative frameworks for concurrent patient and model testing (133), can help expedite the translation of laboratory discoveries to clinical practice. Coclinical trials, which employ the same treatment strategies in patients and their matching patient-derived organoids, are of great interest. This parallel testing approach enables rapid assessment of treatment efficacy and safety and protocol adaptations based on real-time data and supports informed predictions about the success of therapies (134). For a patient population that is as profoundly underrepresented in clinical trials as ILC, the coclinical approach offers an important opportunity to tailor patient-specific therapies and minimize exposure to suboptimal treatments. Figure 2 highlights an integrated framework of preclinical, coclinical, and clinical considerations to guide future ILC-specific study designs.

## Conclusion

In conclusion, although our understanding of the distinct biology of ILC and its differences in clinical presentation and behavior continues to grow, ILC remains underrepresented in clinical trials, and potentially actionable ILC-specific therapeutic targets have not been fully explored. Future trials that are designed to be inclusive of patients with ILC or are specifically targeting pathways of interest in this population hold substantial promise for improving outcomes for patients with this unique histology.

## Conflict of interest

DGS, PTS, RBR, and RJ are members of the Lobular Breast Cancer Alliance (LBCA) Scientific Advisory Board.

## Funding support

- SB by the American Cancer Society Institutional Research Grant pilot award (IRG-23-1156148-27-IRG).
- PTS by the Medical Research Futures Fund (APP2035424).
- RBR by the NIH/NCI Cancer Center Support Grant Administrative Supplement (P30 CA051008-31S1) and BellRinger pilot funds from Georgetown Lombardi.
- RJ by the Maor Foundation and the Melanie Rosay Fund for Breast Cancer Research.

## Supplementary Material

Supplemental data

## Figures and Tables

**Figure 1:**
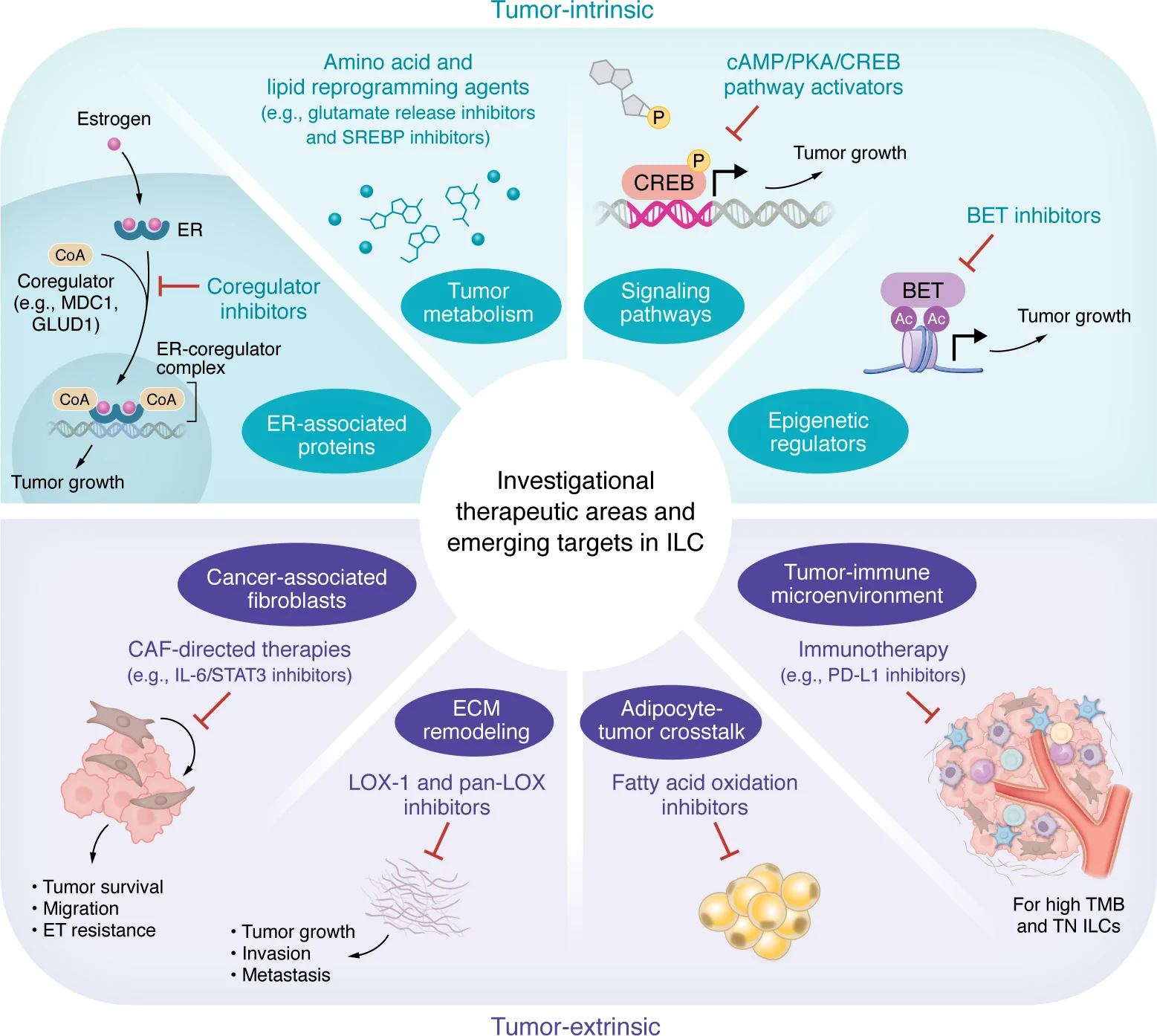
Emerging therapeutic targets in ILC. The schematic summarizes tumor-intrinsic and tumor-extrinsic targets currently under investigation and highlights areas of research that may guide future ILC-specific therapies. TN ILC, triple-negative invasive lobular carcinoma.

**Figure 2:**
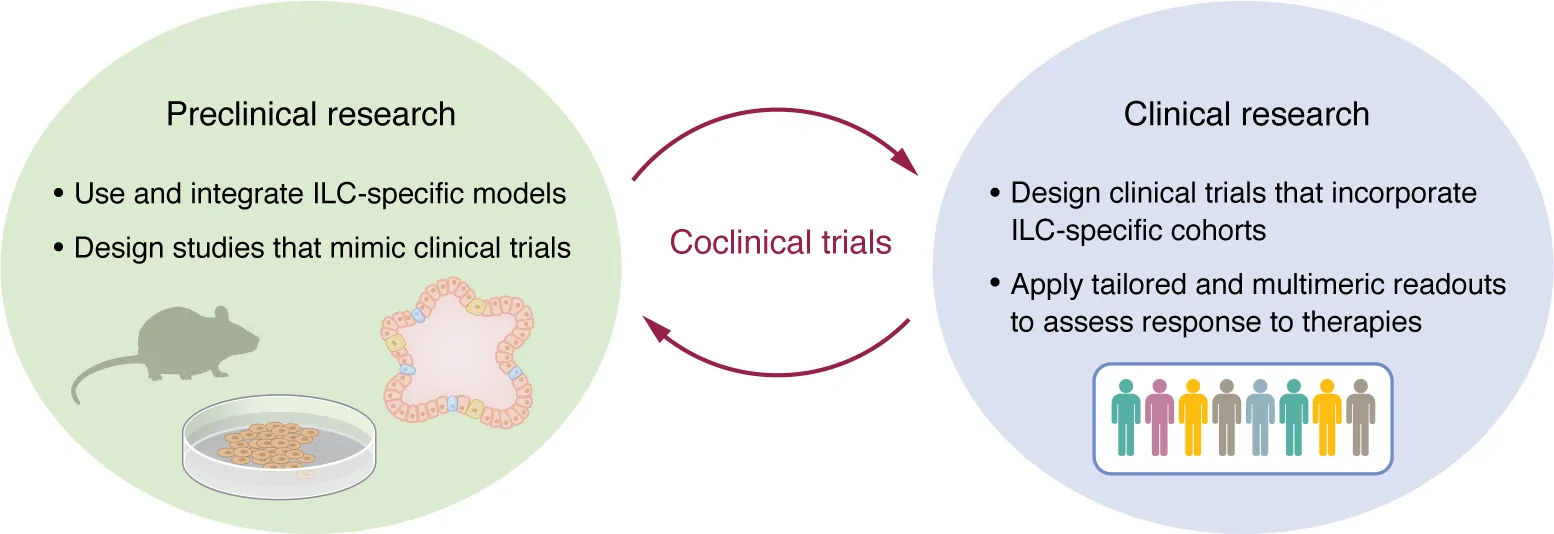
Bridging preclinical and clinical frameworks to accelerate ILC therapeutics. Preclinical testing should use and integrate ILC-specific models (135–137) and aim to mimic clinical trial structure. Future clinical trials incorporating prespecified ILC cohorts, tailored or emerging multimeric readouts, and parallel testing in patients and patient-derived models could expedite the development and evaluation of ILC-targeted therapeutics.

**Table 1:**
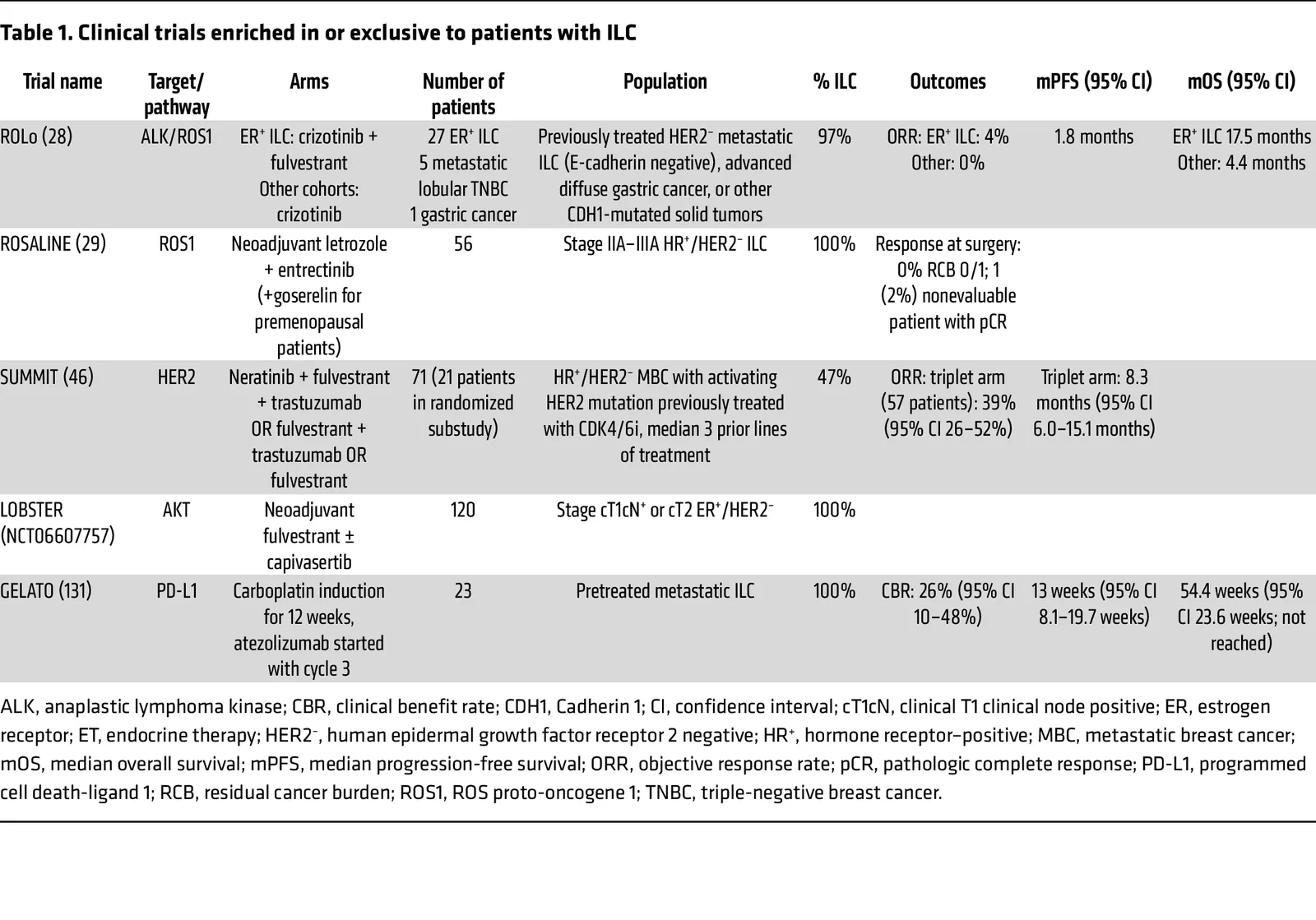
Clinical trials enriched in or exclusive to patients with ILC

## Version 1 10/01/2026

Electronic publication
