# Supplementary material for: Toward targeted therapeutics for lobular breast cancer
Source: J Clin Invest. 2026 Oct 1;136(19):e207629. doi: 10.1172/JCI207629 (PMC13626819; doi:10.1172/JCI207629)
Supplement: Supplementary file 1 [file jci-136-207629-s165.pdf]

Online supplementary material regarding the review manuscript

Toward targeted therapeutics for lobular breast cancer

Kristina A. Fanucci[1,2*], Shaymaa Bahnassy[3*], Arya Mariam Roy[4], Anna Sokolova[5,6], Daniel G. Stover[4], Peter T. Simpson[5], Rebecca B. Riggins[3], Rinath Jeselsohn[1,2]

[1]Department of Medical Oncology, Dana-Farber Cancer Institute, Boston, MA, USA

[2]Harvard Medical School, Boston, MA, USA

[3]Department of Oncology, Lombardi Comprehensive Cancer Center, Georgetown University, Washington, DC, USA

[4]Division of Medical Oncology, Department of Internal Medicine, The Ohio State University, Columbus, OH, USA

[5]UQ Centre for Clinical Research, Faculty of Health, Medicine and Behavioural Sciences, The University of Queensland, Brisbane, Queensland, Australia

[6]Sullivan and Nicolaides Pathology, Brisbane, Queensland, Australia

*These authors contributed equally to this work.

Corresponding author:

Rinath Jeselsohn

Dana-Farber Cancer Institute

450 Brookline Avenue

Boston, MA 02215

Tel: 617-625-3800

Rinath_Jeselsohn@DFCI.HARVARD.EDU

Supplemental table 1. Clinical trials with treatment implications for patients with invasive lobular carcinoma

Trial name	Target/Pathway	Arms	Number of patients	Population	Outcomes	mPFS (HR, 95% CI, p)	mOS (HR, 95% CI, p)
ASPIRE - WI231696 (1)	SRC	Bosutinib + fulvestrant + palbociclib	18	HR+ HER2- MBC progressed on prior CDK4/6i (median prior lines 3.2)	CBR: 50% at 6 months	5.9 months (95% CI 3-10.1)	
SOLAR-1 (2, 3)	PIK3CA	Alpelisib + fulvestrant vs. placebo + fulvestrant	*PIK3CA* mutant cohort: 341	HR+ HER2- ABC with prior treatment with ET	ORR: *PIK3CA* mutant: 26.6% vs 12.8%	*PIK3CA* mutant population: 11.0 (95% CI 7.5-14.5) vs. 5.7 (95% CI 3.7-7.4), (HR=0.65 95% CI 0.50-0.85, p<0.001)	*PIK3CA* mutant: 39.3 (95% CI 34.1-44.9) vs. 31.4 (95% CI 26.8-41.3), (HR=0.86, 95% CI 0.64-1.15, p=0.15)
BYLieve (4)	PIK3CA	Alpelisib + fulvestrant	127	HR+/HER2- ABC with *PIK3CA* mutation after progression on CDK4/6i	ORR: 19% (95% CI 13-28)	8.0 months (95% CI 5.6-8.6)	27.3 months (95% CI 21.3-32.7)
Capitello-291 (5)	AKT	Capivasertib + fulvestrant vs. placebo + fulvestrant	With AKT pathway alteration: 289	HR+ HER2- ABC with progression on prior AI. Up to 2 prior lines of ET and 1 prior line of chemotherapy allowed	ORR: AKT pathway altered: 28.8% vs 9.7%, OR: 3.93 (95%CI 1.93-8.04)	AKT pathway altered: 7.3 vs. 3.1 (HR=0.50; 95% CI 0.38-0.65; p<0.001)	Immature: at 18 months in AKT pathway altered population: 73.2% (95% CI 64.8-80.0) vs 62.9% (95% CI 53.1-72.1), (HR=0.69, 95% CI 0.45-1.05)
INAVO120 (6)	PIK3CA	Inavolisib + palbociclib + fulvestrant vs. placebo + palbociclib + fulvestrant	325	HR+ *PIK3CA*-mutated ABC with progression on or within 12 months of completion of adjuvant ET	ORR: 62.7% (95% CI 54.8-70.2) vs 28.0% (95% CI 21.3-35.6), p<0.001	17.2 vs 7.3 months, (HR=0.42; 95% CI 0.32-0.55)	Immature: 34.0 (95% CI 28.4-44.8) vs. 27.0 (95% CI 22.8-38.7), (HR=0.67, 95% CI 0.48-0.94, p=0.02)
VIKTORIA-1 (PIK3CA-wt) (7)	Pan-PI3K/mTOR	Fulvestrant, fulvestrant + gedatolisib, or fulvestrant + gedatolisib + palbociclib	392	HR+ HER2- PIK3CA-wt ABC with progression on prior CDK4/6 inhibitor		9.3 months fulvestrant + gedatolisib + palbociclib 2.0 months fulvestrant (HR=0.24, 95% CI 0.17-0.35, p<0.001) 7.4 months fulvestrant + gedatolisib vs 2.0 months fulvestrant (HR 0.33, 95% CI 0.24-0.48, p<0.001)	
VIKTORIA-1 (PIK3CA-mutant) (8)	Pan-PI3K/mTOR	Gedatolisib + fulvestrant, alpelisib + fulvestrant, or gedatolisib + palbociclib + fulvestrant	362	HR+ HER2- PIK3CA-wt ABC with progression on prior CDK4/6 inhibitor		11.1 months (gedatolisib + fulvestrant + palbociclib) vs. 5.6 months alpelisib + fulvestrant (HR=0.51, 95% CI 0.37-0.68, p<0.0001); 11.3 gedatolisib + fulvestrant vs. 5.6 months alpelisib + fulvestrant (HR=0.51, 95% CI 0.33-0.79, p<0.0013)	
DESTINY-Breast06 (9)	HER2	Trastuzumab-deruxtecan vs. TPC chemotherapy	713 (HER2-low) 153 (HER2-ultralow)	HR+ HER2-low or HER2-ultralow with 1 or more lines of ET but no prior chemotherapy	ORR: HER2-low: 56.5% (95% CI 51.2-61.7) vs. 32.2% (95% CI 27.4-37.3). HER2-ultralow:	HER2-low: 13.2 (95% CI 11.4-15.2) vs. 8.1 (7.0 vs. 9.0), (HR=0.62, 95% CI 0.52-0.75, p<0.001)	

					61.8% (95% CI 50.0-72.8) vs. 26.3% (95% CI 16.9-37.7)	HER-2 ultralow: 13.2 (95% CI, 9.8-17.3) vs. 8.3 (5.8-15.2), (HR 0.78, 95% CI 0.50-1.21)	
DESTINY-PanTumor01 (10)	HER2	Trastuzumab-deruxtecan	20 patients with breast cancer (20% total trial), 102 patients total	Metastatic solid tumors with activating HER2 mutation, median of 3 prior lines of treatment	ORR: whole population: 29.4% (95% CI ORR: 20.8-39.3)<br>breast: 50% (95% CI 27.2-72.8)	Whole population: 5.4 months (95% CI 2.7–7.1)	Whole population: 10.9 months (95% CI 8.3-14.9)
RADICAL (11)	FGFR1, 2, and 3	AZD4547 + letrozole or anastrozole	52 (4 with *FGFR* amp)	ER+ MBC resistant to ET	ORR: 10%	3.01 months (95% CI 2.4-6)	
RAGNAR (12)	pan-FGFR	Erdafitinib	16	MBC who exhausted SOC with prespecified *FGFR*1-4 alterations, median 5 prior lines of treatment	ORR: 31% (95% CI 11-59)	5.7 months	8.9 months
Debio 1347 (13)	FGFR	Debio 1347	58	Advanced solid tumors with *FGFR* alteration (54% *FGFR* amp), breast cancer (21%)	ORR: 0% in patients with breast cancer		
EMERALD (14, 15)	ER	Elacestrant vs. SOC ET	477, 228 with *ESR1* mutation	ER+/HER2- ABC with 1-2 prior lines of ET including CDK4/6i, up to 1 prior line of chemotherapy	ORR: in patients with *ESR1* mutation: 7.1% (95% CI 2.6-14.7) vs. 4.7% (95% CI 1.3 vs. 11.5), p=0.499	In patients with *ESR1* mutation: 3.65 months (HR=0.65, 95% CI 0.03-23.95 vs. 2.07, 95% CI 0.03-17.12, p=0.0049);<br>In patients with ≥12 months prior CDK4/6i treatment 8.6 vs. 1.9 months (HR=0.41, 95% CI 0.26-0.63)	
evERA (16)	ER	Giredestrant + everolimus vs. SOC ET + everolimus	373, 207 *ESR1* mutant population	ER+/HER2-, 1-2 prior lines of ET, no prior chemotherapy, prior CDK4/6i	ORR: 26.6% vs 13.8% (95% CI 0.48-24.7)	*ESR1* mutant population: HR=0.38, (95% CI 0.27-0.54), 10 months (95% CI 8.08-12.94) vs. 5.5 (95% CI 3.75-5.62, P<0.0001)	Immature: NR (95% CI 20.17-NE) vs 21.03 (95% CI 14.78-26.87), (HR=0.62, 95% CI 0.38-1.02, p=0.0566
EMBER-3 (17)	ER	SOC ET vs. imlunestrant vs. imlunestrant + abemaciclib	874, 256 *ESR1* mutation	ER+ HER2- ABC, progressed on AI +/-CDK4/6i		Imlunestrant vs. SOC in *ESR1* mutant: 5.5 months (95% CI 3.9-7.4) vs 3.8 (95% CI 3.7-5.5)<br>Without *ESR1* mutation: 5.6 months vs. 5.7 months (HR 1.00, 95% CI 0.79-1.27)<br><br>Imlunestrant + abemaciclib vs. imlunestrant: 9.4 months (95% CI 7.5-11.9) vs. 5.5 months (95% CI 3.8-5.6),	Immature: *ESR1* mutant: OS at 18 months imlunestrant vs. SOC: 77% (95% CI 67.4-84.1) vs. 58.6% (95% CI 47.2-68.3), (HR=0.55, 95% CI 0.35-0.86, p=0.008)<br>Without *ESR1* mutation: HR 0.87 (95% CI 0.54-1.4)

						(HR= 0.57, 95% CI 0.44-0.73, p<0.001)	
SERENA-6 (18)	ER	Continued CDK4/6i with switch to camizestrant vs. continuation of SOC ET	315	First line CDK4/6i + ET who develop *ESR1* mutation	ORR: 7.0% vs 1.9% HR=5.11 (0.63-10.46), p=0.024	16.0 (95% CI 12.7-18.2) vs. 9.2 (95% CI 7.2-9.5) months, (HR=0.44, 95% CI 0.31-0.60, P<0.0001	Immature: HR 0.91 (95% CI: 0.48-1.73)
ICARUS-BREAST01 (19)	HER3	Patritumab deruxtecan	99	HR+ HER2- MBC progressed on CDK4/6i + ET and one line chemotherapy	ORR: 53.5% (90% CI 44.8-62.1%)	9.2 months (95% CI 8.0-12.8)	

ABC, advanced breast cancer; AI, aromatase inhibitor; CBR, clinical benefit rate; CDK4/6i, cyclin-dependent kinases 4/6 inhibitor; CI, confidence interval; ER, estrogen receptor; ET, endocrine therapy; HR, hazard ratio; HR+, hormone receptor-positive; ET, endocrine therapy; ILC, invasive lobular carcinoma; MBC, metastatic breast cancer; mOS, median overall survival; mPFS, median progression-free survival; NE, not estimable; NR, not releasable; OR, odds ratio; ORR, objective response rate; OS, overall survival; pCR, pathologic complete response; PD-L1, programmed death-ligand 1; SOC, standard of care; TNBC, triple-negative breast cancer; TPC, treating physician's choice; WT, wildtype

Supplemental references

1. Mainor CB, et al. A phase I trial of palbociclib (palbo) and bosutinib (bos) with fulvestrant (fulv) in patients (pts) with hormone receptor–positive, HER2-negative (HR+/HER2-) metastatic breast cancer (MBC) refractory to an aromatase inhibitor (AI) and a CDK4/6 inhibitor (CDK4/6i). *J Clin Oncol.* 2023;41(16_suppl):1079-1079.
2. Andre F, et al. Alpelisib for PIK3CA-mutated, hormone receptor-positive advanced breast cancer. *N Engl J Med.* 2019;380(20):1929-1940.
3. Andre F, et al. Alpelisib plus fulvestrant for PIK3CA-mutated, hormone receptor-positive, human epidermal growth factor receptor-2-negative advanced breast cancer: Final overall survival results from Solar-1. *Ann Oncol.* 2021;32(2):208-217.
4. Rugo HS, et al. Alpelisib plus fulvestrant in PIK3CA-mutated, hormone receptor-positive advanced breast cancer after a CDK4/6 inhibitor (BYLieve): one cohort of a phase 2, multicentre, open-label, non-comparative study. *Lancet Oncol.* 2024;25(12):e629-e638.
5. Turner NC, et al. Capivasertib in hormone receptor-positive advanced breast cancer. *N Engl J Med.* 2023;388(22):2058-2070.
6. Jhaveri KL, et al. Overall survival with inavolisib in PIK3CA-mutated advanced breast cancer. *N Engl J Med.* 2025;393(2):151-161.
7. Hurvitz SA, et al. VIKTORIA-1 trial of gedatolisib plus fulvestrant with or without palbociclib in hormone receptor-positive/HER2-/PIK3CA wild-type advanced breast cancer. *J Clin Oncol.* 2026;44(12):1108-1119.

8. Hurvitz SA, et al. A randomized, open-label, phase 3 study of gedatolisib + fulvestrant ± palbociclib vs standard of care in HR+/HER2−/PIK3CA-mutant (MT) advanced breast cancer (VIKTORIA-1 Study 2) [abstract]. *J Clin Oncol.* 2026;44(17_suppl):LBA1008.
9. Bardia A, et al. Trastuzumab deruxtecan after endocrine therapy in metastatic breast cancer. *N Engl J Med.* 2024;391(22):2110-2122.
10. Li BT, et al. Trastuzumab deruxtecan in patients with solid tumours harbouring specific activating HER2 mutations (DESTINY-PanTumor01): An international, phase 2 study. *Lancet Oncol.* 2024;25(6):707-719.
11. Coombes RC, et al. Results of the phase IIa RADICAL trial of the FGFR inhibitor AZD4547 in endocrine resistant breast cancer. *Nat Commun.* 2022;13(1):3246.
12. Carranza O, et al. Efficacy and safety of erdafitinib in adults with breast cancer and prespecified fibroblast growth factor receptor alterations in the phase 2 open-label, single-arm RAGNAR trial. *J Clin Oncol.* 2024;42(16_suppl):1088.
13. Voss MH, et al. A phase I, open-label, multicenter, dose-escalation study of the oral selective FGFR inhibitor Debio 1347 in patients with advanced solid tumors harboring FGFR gene alterations. *Clin Cancer Res.* 2019;25(9):2699-2707.
14. Bardia A, et al. Elacestrant in ER+, HER2- metastatic breast cancer with ESR1-mutated tumors: Subgroup analyses from the phase III EMERALD trial by prior duration of endocrine therapy plus CDK4/6 inhibitor and in clinical subgroups. *Clin Cancer Res.* 2024;30(19):4299-4309.
15. Bidard FC, et al. Elacestrant (oral selective estrogen receptor degrader) versus standard endocrine therapy for estrogen receptor-positive, human epidermal growth factor

receptor 2-negative advanced breast cancer: Results from the randomized phase III EMERALD trial. *J Clin Oncol.* 2022;40(28):3246-3256.

16. Mayer E, et al. Giredestrant (GIRE), an oral selective oestrogen receptor (ER) antagonist and degrader, + everolimus (E) in patients (pts) with ER-positive, HER2-negative advanced breast cancer (ER+, HER2– aBC) previously treated with a CDK4/6 inhibitor (i): Primary results of the phase III evERA BC trial. *Ann Oncol.* 2025;36:S1561-S1562.

17. Jhaveri KL, et al. Imlunestrant with or without abemaciclib in advanced breast cancer. *N Engl J Med.* 2025;392(12):1189-1202.

18. Bidard FC, et al. First-line camizestrant for emerging ESR1-mutated advanced breast cancer. *N Engl J Med.* 2025;393(6):569-580.

19. Pistilli B, et al. Patritumab deruxtecan in HR+HER2- advanced breast cancer: A phase 2 trial. *Nat Med.* 2025;31(10):3492-3503.
